# Tomato *SlMKK2* and *SlMKK4* contribute to disease resistance against *Botrytis cinerea*

**DOI:** 10.1186/1471-2229-14-166

**Published:** 2014-06-15

**Authors:** Xiaohui Li, Yafen Zhang, Lei Huang, Zhigang Ouyang, Yongbo Hong, Huijuan Zhang, Dayong Li, Fengming Song

**Affiliations:** 1 National Key Laboratory for Rice Biology, Institute of Biotechnology, Zhejiang University, Hangzhou, Zhejiang 310058, China

**Keywords:** Tomato (*Solanum lycopersicum*), MAPK cascade, MPK kinase, SlMKK2/SlMKK4, *Botrytis cinerea*, Defense response

## Abstract

**Background:**

Mitogen-activated protein kinase (MAPK) cascades are highly conserved signaling modules that mediate the transduction of extracellular stimuli via receptors/sensors into intracellular responses and play key roles in plant immunity against pathogen attack. However, the function of tomato MAPK kinases, SlMKKs, in resistance against *Botrytis cinerea* remains unclear yet.

**Results:**

A total of five *SlMKK* genes with one new member, *SlMKK5*, were identified in tomato. qRT-PCR analyses revealed that expression of *SlMKK2* and *SlMKK4* was strongly induced by *B. cinerea* and by jasmonic acid and ethylene precursor 1-amino cyclopropane-1-carboxylic acid. Virus-induced gene silencing (VIGS)-based knockdown of individual *SlMKKs* and disease assays identified that *SlMKK2* and *SlMKK4* but not other three *SlMKKs* (*SlMKK1*, *SlMKK3* and *SlMKK5*) are involved in resistance against *B. cinerea*. Silencing of *SlMKK2* or *SlMKK4* resulted in reduced resistance to *B. cinerea*, increased accumulation of reactive oxygen species and attenuated expression of defense genes after infection of *B. cinerea* in tomato plants. Furthermore, transient expression of constitutively active phosphomimicking forms *SlMKK2*^*DD*^ and *SlMKK4*^*DD*^ in leaves of *Nicotiana benthamiana* plants led to enhanced resistance to *B. cinerea* and elevated expression of defense genes.

**Conclusions:**

VIGS-based knockdown of *SlMKK2* and *SlMKK4* expression in tomato and gain-of-function transient expression of constitutively active phosphomimicking forms *SlMKK2*^*DD*^ and *SlMKK2*^*DD*^ in *N. benthamiana* demonstrate that both SlMKK2 and SlMKK4 function as positive regulators of defense response against *B. cinerea*.

## Background

During their life time, plants always suffer from invasion of potential pathogenic microorganisms in the environment. To defend themselves against pathogen attack, plants have evolved a sophisticated immune system [1-3]. Two types of innate immune responses, which are precisely regulated upon infection from different types of pathogens, have been recognized in plants so far. The first innate immune response is the pathogen-associated molecular pattern (PAMP)-triggered immunity (PTI), which is activated by a number of PAMPs such as flagellin, EF-Tu and chitin [4-6]. The other one is the effector-triggered immunity (ETI), which is modulated by recognition of pathogen-derived avirulence effectors by plant R proteins [7,8]. Once initiation of the innate immune responses, plant cells can often trigger a series of signaling events that lead to diverse cellular responses including changes in ion fluxes, synthesis of the defense-related hormones, transcriptional reprogramming, production of reactive oxygen species (ROS), and a localized form of programmed cell death (PCD) referred to as the hypersensitive response (HR) [9]. These signals are translated from outside into plant cells by some conserved signal molecules and trigger plant downstream immune responses [10].

Mitogen-activated protein kinase (MAPK) cascades are highly conserved signaling modules downstream of receptors/sensors that transduce extracellular stimuli into intracellular responses [11]. The MAPK cascade comprises three functional protein kinases, i.e. MAPK kinase kinases (MAPKKKs), MAPK kinases (MAPKKs) and MAPKs. Upon perception of the environmental signals by the membrane-localized receptor-like protein kinases, MAPKKKs activate via phosphorylation their downstream MAPKKs, which in turn further phosphorylate MAPKs [12]. The input signal can be amplified through the MAPK cascade to modify a set of specific downstream target proteins by the way of phosphorylation [12]. In *Arabidopsis thaliana*, 80 MAPKKKs, 10 MAPKKs and 20 MAPKs have been recognized [13,14] and some of them have been studied extensively for their functions in plant immunity. Two entire Arabidopsis MAPK cascades, MEKK1-MKK4/MKK5-MPK3/MPK6 and MEKK1-MKK1/2-MPK4, have been established through genetic and biochemical studies and have been shown to act as positive or negative regulators of signaling pathways involved in immune responses such as PTI and ETI [11,15,16]. The components of the MEKK1-MKK4/MKK5-MPK3/MPK6 cascade can be activated rapidly upon treatment with some of PAMPs such as flg22, a peptide PAMP derived from bacterial flagellin [17]. Knockout/knockdown of individual component in this MAPK cascade normally results in increased disease susceptibility to a range of pathogens including *Pseudomonas syringae* pv. *tomato* DC3000 and *Botrytis cinerea*[18-20], whereas transient or stable expression of constitutively active phosphomimic MKK4/MKK5 in Arabidopsis leaves or transgenic plants leads to enhanced resistance to bacterial and fungal pathogens and activated defense responses including expression of defense genes, generation of ROS, accumulation of camalexin and appearance of HR-like cell death [17,19,21-24]. By contrast, the MEKK1-MKK1/2-MPK4 cascade plays both positive and negative roles in regulating plant defense. The *mekk1*, *mkk1*/*mkk2* double and *mpk4* plants exhibit constitutively activated defense responses, i.e. accumulation of ROS, elevated level of salicylic acid (SA) expression of defense genes and HR, and display enhanced resistance to a range of pathogens [25-31]. Genetic, molecular and biochemical studies have also identified a number of components of the MAPK cascades from other plants such as tobacco and rice, which play important roles in regulating disease resistance responses against different types of pathogens (for reviews, see [11,15,16,32,33].

In tomato, a total of 16 putative SlMPKs were identified at genome-wide level [34] and some of them have been functionally characterized for their possible roles in regulating defense response against biotic stresses. SlMPK1, SlMPK2 and SlMPK3 were shown to participate in *Cf-4*/*Avr4-* and *Pto*/*AvrPto*-induced HR and in defense response against *Ralstonia solanacearum* and insect attack [35-39]. SlMPK4, a homolog of Arabidopsis MPK4 that is a negative regulator of immunity [25], was shown to be required for resistance against *B. cinerea*[40]. SlMKK2 and SlMKK4, two out of four tomato SlMKKs identified, can phosphorylate SlMPK1, SlMPK2 and SlMPK3 and induce HR-like cell death when overexpressed in tomato leaves, unraveling a possible MAPK cascade in defense response against *P. syringae* pv. *tomato*[35,41,42]. Biochemical evidence has revealed that two leucines in the D-site of SlMKK2 are critical to interact with SlMPK3 and PCD elicitation [42]. Two MAPKKKs (MAPKKKα and MAPKKKϵ) have been shown to function as positive regulators of *Pto*-mediated signal transduction [43,44]. Recently, it was found that a tomato 14-3-3 protein TFT7 can interact with both SlMAPKKKα and SlMKK2 and may coordinately recruit SlMAPKKKα and SlMKK2 for efficient signaling leading to PCD [45,46].

Despite of extensive studies on the MAPK cascades in immune response in tomato, little is known about the functions of these MAPK cascades in defense response against necrotrophic fungal pathogens such as *B. cinerea*. In the present study, we performed functional analyses using virus-induced gene silencing (VIGS) approach of *SlMKKs* in resistance against *B. cinerea* and found that both SlMKK2 and SlMKK4 act as positive regulators of defense response against this necrotrophic fungal pathogen.

## Results

### Identification of tomato SlMKKs

**Figure 1:**
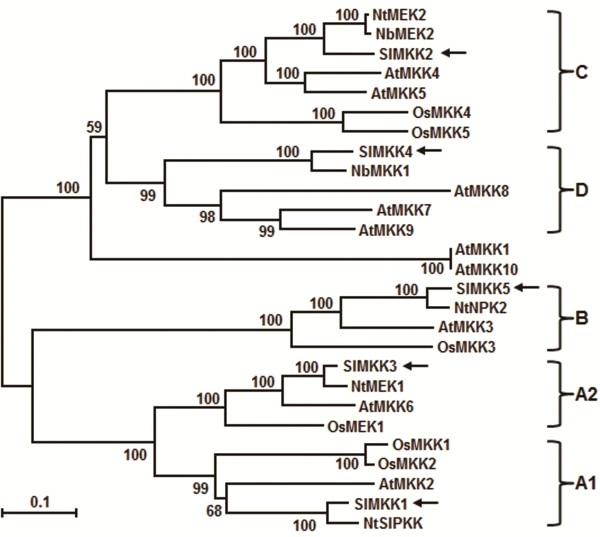
**Phylogenetic tree of SlMKKs with other plant MKKs.** Phylogenetic tree was constructed by neighbour-joining method using MEGA program version 6.05. SlMKKs in the tree are indicated by arrows and the five groups of plant MKKs are also indicated at right of the tree. Plant MKK proteins used and their GenBank accessions are as follows: AtMKK1 (NP_194337), AtMKK2 (NP_001031751), AtMKK3 (NP_198860), AtMKK4 (NP_175577), AtMKK5 (NP_188759), AtMKK6 (NP_200469), AtMKK7 (NP_173271), AtMKK8 (NP_187274), AtMKK9 (NP_177492), AtMKK10 (NP_174510), NbMEK2 (BAG31944), NbMKK1 (BAE95414), NtMEK1 (CAC24705), NtMEK2 (BAE97401), NtNPK2 (BAA06731), NtSIPKK (AAF67262), OsMEK1 (NP_001043164), OsMKK1 (ABP88102), OsMKK2 (NP_001056806), OsMKK3 (ABN50916), OsMKK4 (NP_001048341), OsMKK5 (BAD35809), SlMKK1 (NP_001234744), SlMKK2 (NP_001234588), SlMKK3 (NP_001234591), SlMKK4 (NP_001234595), SlMKK5 (XP_004234320).

Four *SlMKKs*, *SlMKK1-4*, have previously been identified from tomato through searching expressed sequence tags in the TIGR tomato gene index using the NtMEK2 amino acid sequence as a query [41]. In searches against the tomato genome sequence database (http://solgenomics.net/), we identified one more putative SlMKKs and named as SlMKK5, which is predicted to locus Solyc03g019850. No full-length cDNA was identified in the tomato genome sequence database but an Expressed Sequence Tag (FS196940) was obtained in GenBank database, indicating that the *SlMMK5* gene is normally expressed in tomato plants. This is further supported by our cloning and sequencing of the coding sequence of *SlMKK5*, which encodes a protein of 515 aa, larger than those of SlMKK1-4 (335–359 aa). Phylogenetic tree analysis revealed that SlMKK5, belonging to Group B of plant MKKs [13], is much close to Arabidopsis AtMKK3 and tobacco NtNPK2, showing 76-93% of identity at amino acid level and also shows 26-37% of identity to other Arabidopsis MKKs (Figure 1). Therefore, it is likely that there are five SlMKKs in tomato genome and each of tomato SlMKKs falls into one group of plant MKKs identified so far.

### Expression of *SlMKKs* induced by *B. cinerea* infection and phytohormone treatment

**Figure 2:**
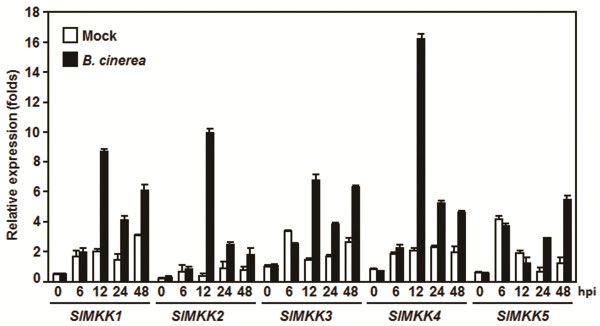
**Expression patterns of *SlMKKs *after inoculation with *Botrytis cincerea*.** Tomato plants were inoculated by foliar spraying with spore suspension (2 × 10^5^ spores/mL) of *B. cinerea* and leaf samples were collected at time points as indicated. Gene expression was analyzed by qRT-PCR and relative expression levels were calculated by comparing with the corresponding values at 0 hr (as a control) after inoculation. Data presented are the means ± SD from three independent experiments and different letters above the columns indicate significant differences at *p* < 0.05 level.

To explore the possible involvement of SlMKKs in defense response against *B. cinerea*, we first analyzed the expression changes of *SlMKKs* after infection with *B. cinerea*. As shown in Figure 2, all five *SlMKKs* were induced upon infection of *B. cinerea* but showed different expression dynamic patterns. Generally, as compared with those in the mock-inoculated plants, the expression of *SlMKK1-4* was induced significantly with peaks at 12 hr and thereafter declined during 24–48 hr after infection with *B. cinerea* (Figure 2). Specifically, the expressions of *SlMKK2* and *SlMKK4* in *B. cinerea*-infected plants showed approximately 45 and 8 folds of increases over those in the mock-inoculated plants at 12 hr after inoculation (Figure 2). The expressions of *SlMKK1* and *SlMKK3* exhibited 3–4 folds of increases at 12 hr after infection of *B. cinerea*. However, unlike the expression dynamics of *SlMKK1-4*, the expression of *SlMKK5* was not induced significantly during the early stage of infection but showed an increase after 24 hr, showing 5 folds of increases (Figure 2). These results indicate that the tomato *SlMKKs* respond to infection of *B. cinerea* with different dynamics and magnitude of expression and that *SlMMK2* and *SlMKK4* have stronger induction of expression upon *B. cinerea* infection.

**Figure 3:**
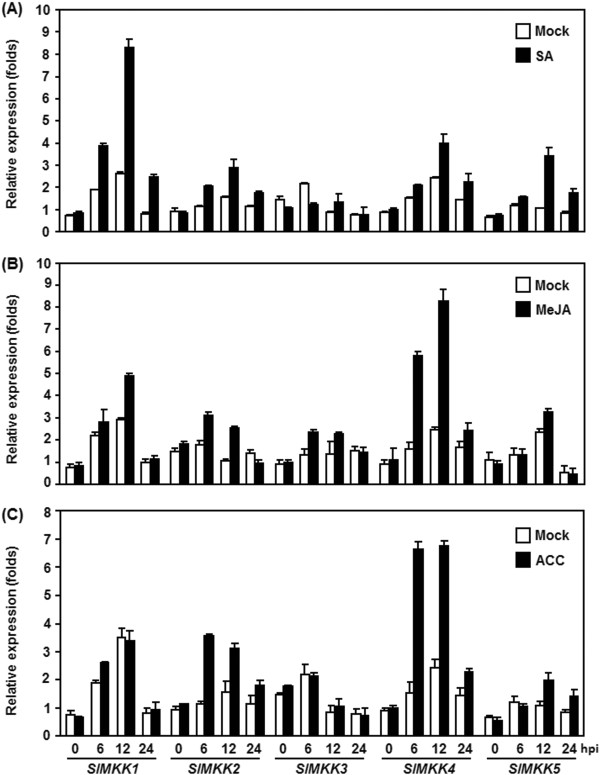
**Expression patterns of *SlMKKs *after treatment with defense signaling hormones.** Tomato plants were treated by foliar spraying of SA **(A)**, MeJA **(B)** or ACC **(C)** and leaf samples were collected at time points as indicated. Gene expression was analyzed by qRT-PCR and relative expression levels were calculated by comparing with the corresponding values at 0 hr (as a control) after treatment. Data presented are the means ± SD from three independent experiments and different letters above the columns indicate significant differences at *p* < 0.05 level.

We further examined the dynamics of *SlMKKs* expressions in tomato plants after treatment with SA, methyl jasmonate (MeJA) and 1-amino cyclopropane-1-carboxylic acid (ACC) [a precursor of ethylene (ET)]. As shown in Figure 3, different dynamics of expression patterns for *SlMKKs* were observed in response to these defense signaling hormones. In SA-treated plants, expression of *SlMKK1* and *SlMKK5* was significantly increased by 2–3 folds over that in the control plants, while expressions of *SlMKK2*, *SlMKK3* and *SlMKK4* were not affected markedly by SA (Figure 3A). In MeJA- or ACC-treated plants, expression of *SlMKK4* was strongly induced by both MeJA and ACC, reaching 3–4 folds of increased at 6 hr after treatment (Figure 3B and C). *SlMKK2* was also induced by MeJA and ACC, its expression level showed an increase of 3 folds at 6 hr after ACC treatment and exhibited an increase of 2.5 folds at 12 hr after MeJA treatment (Figure 3B and C). However, the expressions of *SlMKK1*, *SlMKK3* and *SlMKK5* were not affected by MeJA and ACC during our experimental period. Therefore, it is clear that the tomato *SlMKKs* also respond with different expression patterns to SA, JA and ET, three well-known defense signaling hormones.

### Silencing of *SlMKK2*/*SlMKK4* resulted in reduced resistance to *B. cinerea*

To examine the possible involvement of *SlMKKs* in disease resistance against *B. cinerea*, we performed functional analyses on all five *SlMKKs* identified by VIGS approach through comparing the phenotype of disease caused by *B. cinerea* between individual *SlMKK*-silenced plants with control plants. For this purpose, specific fragment for each *SlMKK* gene was chosen to generate VIGS construct and standard VIGS procedure with a phytoene desaturase (PDS) construct as an indicative for VIGS efficiency of each experiment was performed on 2-week-old plants [47]. Only when >90% of the *PDS* construct-infiltrated plants showed bleaching phenotype, the VIGS construct of interest gene-infiltrated plants were used for experiments. The silencing efficiency and specificity for each *SlMKK* gene was determined by qRT-PCR analyzing the transcript level of the target *SlMKK* gene and other four *SlMKK* genes in the TRV-target *SlMKK*-infiltrated plants. In our experiment condition, when compared with those in the TRV-GUS-infiltrated plants, the transcript level of the target *SlMKK* gene was significantly reduced whereas the transcript levels of the other *SlMKK* genes were comparable in the TRV-target *SlMKK*-silenced plants (data not shown). Overall, the silencing efficiency for a target *SlMKK* gene was approximately 70-75% (data not shown). Therefore, the silencing efficiencies and specificity for each *SlMKK* gene were satisfied for further experiments.

**Figure 4:**
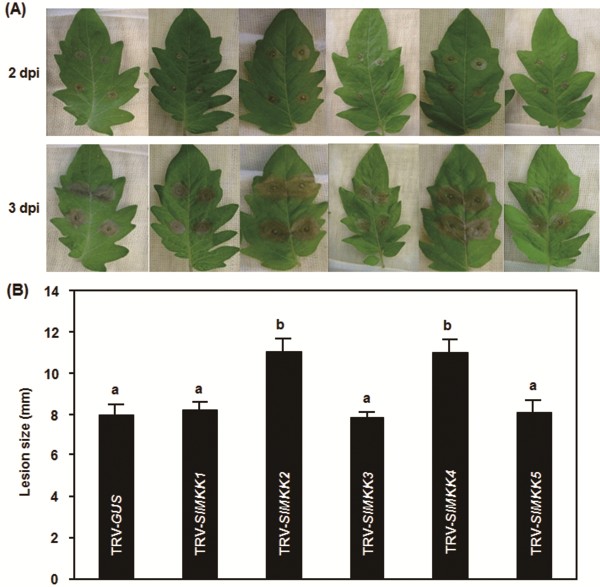
**Disease phenotype of *SlMKKs*-silenced plants after inoculation with *B. cinerea.*** Disease symptom **(A)** and lesion size **(B)** in selected leaves of the TRV-SlMKKs- and TRV-GUS-infiltrated plants in detached leaf inoculation assays. *Botrytis* inoculation was done by dropping spore suspension (1 × 10^5^ spores/mL) on detached leaves of tomato plants and lesion sizes were measured at 3 days after inoculation on a minimum of 20 leaves in each experiment. At least ten leaves from ten individual silenced or control plants were used for each experiment. Data presented in **(B)** are the means ± SD from three independent experiments and different letters above the columns indicate significant differences at *p* < 0.05 level.

**Figure 5:**
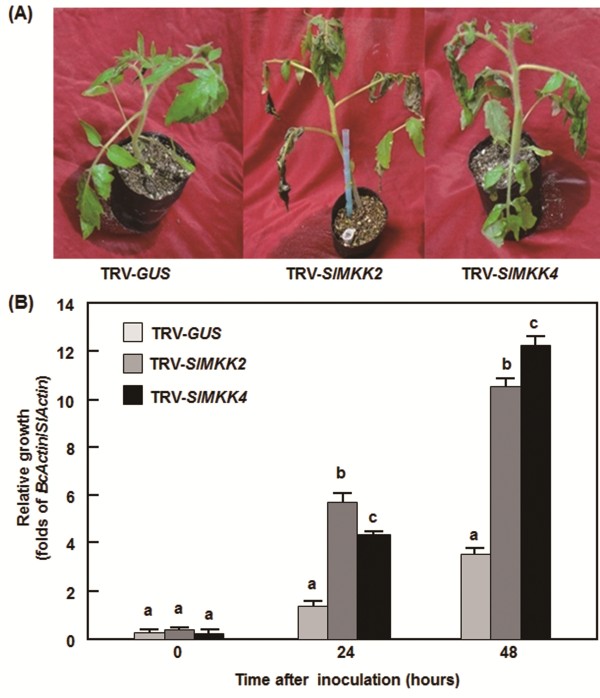
**Silencing of *SlMKK2 *and *SlMKK4 *increased severity of disease caused by *B. cinerea.*** Disease phenotype **(A)** and fungal growth **(B)** on the TRV-SlMKK2/4- and TRV-GUS-infiltrated plants in whole plant inoculation assays. *Botrytis* inoculation was done by foliar spraying with spore suspension (2 × 10^5^ spores/mL) onto leaves of whole plants. Fungal growth *in planta* was assumed by analyzing the transcript levels of *BcActinA* gene by qRT-PCR using *SlActin* as an internal control at the indicated time points after inoculation. At least ten leaves from ten individual silenced or control plants were used for each experiment. Data presented in **(B)** are the means ± SD from three independent experiments and different letters above the columns indicate significant differences at *p* < 0.05 level.

To investigate the roles of *SlMKKs* in disease resistance against *B. cinerea*, we used two different strategies, detached leaf disease assays for fast evaluation and whole plant disease assays for confirmation, to compare the disease phenotype and *in planta* fungal growth in the TRV-target SlMKK-infiltrated plants with those in the TRV-GUS-infiltrated plants. In detached leaf disease assays, typical disease lesions were observed 2 days post inoculation (dpi) (Figure 4A). The lesions on leaves from the TRV-SlMKK2- or TRV-SlMKK4-infiltrated plants were larger than that in the TRV-GUS-infiltrated plants at 2 dpi and began to merge into large necrotic areas at 3 dpi (Figure 4A), showing an approximately 40% of increase in lesion size over those on leaves from the TRV-GUS-infiltrated control plants (Figure 4B). The lesions on leaves from the TRV-SlMKK1-, TRV-SlMKK3- and TRV-SlMKK5-infiltrated plants were similar to that in the TRV-GUS-infiltrated plants (Figure 4A and B). Further whole plant disease assays were carried out to confirm the disease phenotype observed in the TRV-SlMKK2- and TRV-SlMKK4-infiltrated plants. In the whole plant disease assays, the TRV-SlMKK2- and TRV-SlMKK4-infiltrated plants along with the TRV-GUS-infiltrated plants were inoculated by spraying with spore suspension of *B. cinerea* and disease phenotype and *in planta* fungal growth were observed and analyzed, respectively. As shown in Figure 5A, the TRV-GUS-infiltrated control plants displayed slight disease, whereas the TRV-SlMKK2- or TRV-SlMKK4-infiltrated plants showed severe diseases, showing large necrotic areas and maceration or wilting of full leaves at 5 dpi. Analysis of the transcript for the *B. cinerea* actin gene *BcActinA* as an indicator of the rate of fungal growth *in planta* further confirmed that the TRV-SlMKK2- and TRV-SlMKK4-infiltrated plants showed reduced resistance to *Botrytis* infection than the TRV-GUS-infiltrated control plants (Figure 5B). Growth of *B. cinerea* in leaf tissues of the TRV-SlMKK2- or TRV-SlMKK4-infiltrated plants had three times higher than those in the TRV-GUS-infiltrated control plants at 24 and 48 hr after inoculation (Figure 5B), indicating much fungal growth in the *SlMKK2-* or *SlMKK4-*silenced plants. These data demonstrate that knockdown of the *SlMKK2* or *SlMKK4* resulted in reduced resistance to *B. cinerea* and thus both *SlMKK2* and *SlMKK4* are required for resistance against *B. cinerea*.

### Silencing of *SlMKK2*/*SlMKK4* attenuated defense response against *B. cinerea*

**Figure 6:**
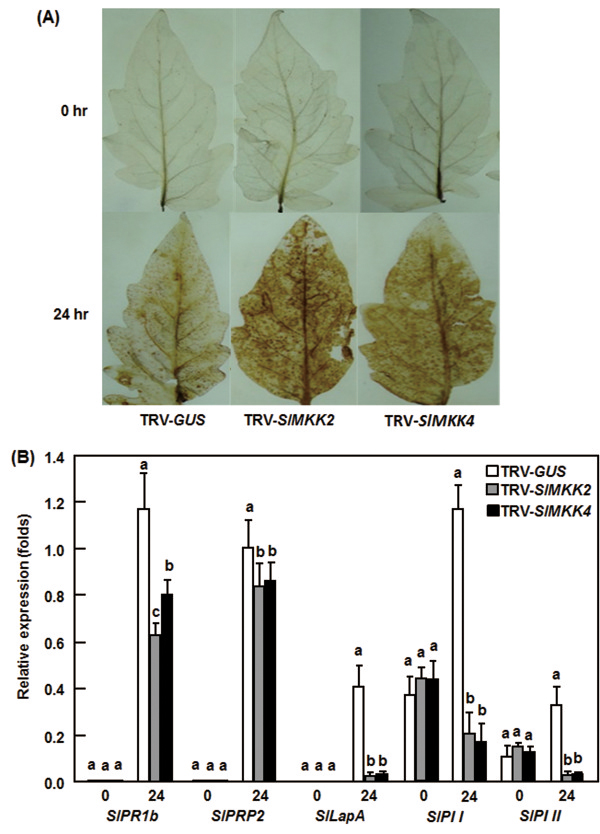
**Silencing of *SlMKK2 *and *SlMKK4 *attenuated *B. cinerea*-induced defense response.** Accumulation of H_2_O_2_**(A)** and expression of defense-related genes **(B)** in the TRV-SlMKK2/4- and TRV-GUS-infiltrated plants in whole plant inoculation assays. Accumulation of H_2_O_2_ was detected by DAB staining at 24 hr after inoculation. Gene expression was analyzed by qRT-PCR and relative expression levels were calculated by comparing with the corresponding values at 0 hr (as a control) after inoculation. Data presented in **(B)** are the means ± SD from three independent experiments and different letters above the columns indicate significant differences at *p* < 0.05 level.

To elucidate the physiological and molecular mechanisms involved in the reduced resistance in the *SlMKK2*- or *SlMKK4*-silenced plants, we analyzed and compared the accumulation of ROS such as H_2_O_2_ and expression of defense genes before and after infection with *B. cinerea* between the *SlMKK2*- or *SlMKK4*-silenced plants and the control plants. ROS has been demonstrated to play important roles in susceptible response of plants to infection from necrotrophic fungal pathogens, e.g. *B. cinerea*, especially the ROS accumulated during late stage of infection, which directly benefits the growth of the invaded fungus [48]. No difference in accumulation of H_2_O_2_, as detected by DAB staining, was observed in leaves of the TRV-SlMKK2-, TRV-SlMKK4- and TRV-GUS-infiltrated plants without infection of *B. cinerea* (Figure 6A), indicating that silencing of *SlMKK2* or *SlMKK4* itself did not affect the generation and accumulation of H_2_O_2_ in tomato plants. After infection with *B. cinerea*, significant accumulation of H_2_O_2_, shown as brown precipitates in leaves, was detected in leaves of the TRV-SlMKK2-, TRV-SlMKK4- and TRV-GUS-infiltrated plants (Figure 6A). However, the leaves from the TRV-SlMKK2- and TRV-SlMKK4-infiltrated plants showed consistent increase in intensity of the stained areas as compared with the TRV-GUS-infiltrated plants after infection of *B. cinerea* (Figure 6A). These data indicate that silencing of *SlMKK2* or *SlMKK4* accelerates the generation and accumulation of H_2_O_2_ upon infection of *B. cinerea*.

We next analyzed the expression of representative marker genes regulated by the JA/ET- and SA-mediated defense signaling pathways, respectively, to explore the possible molecular mechanism associated with the reduced *B. cinerea* resistance in *SlMKK2*- and *SlMKK4*-silenced plants. For this purpose, two marker genes, *SlPRP2* and *SlPR1b*, regulated by the SA-mediated signaling pathway [49], and another three marker genes, *SlLapA*, *SlPI I* and *SlPI II*, regulated by the JA/ET-mediated signaling pathway [49], were chosen to compare their expression changes in the TRV-SlMKK2- or TRV-SlMKK4-infiltrated plants with those in the TRV-GUS-infiltrated plants. No significant difference in expression of the four defense genes examined was observed in the TRV-SlMKK2-, TRV-SlMKK4- or TRV-GUS-infiltrated plants without infection of *B. cinerea* (Figure 6B), indicating that silencing of *SlMKK2* or *SlMKK4* did not affect the expression of defense genes in tomato plants under normal healthy condition. As compared with those in the mock-inoculated plants, the expression levels of *SlPRP2* and *SlPR1b* increased significantly after infection with *B. cinerea*; however, the expression levels in the TRV-SlMKK2- and TRV-SlMKK4-infiltrated plants were reduced to some extents as compared with those in the TRV-GUS-infiltrated plants (Figure 6B). Similarly, infection of *B. cinerea* also induced significantly the expression of *SlLapA*, *SlPI I* and *SlPI II* (Figure 6B); however, the expression levels of *SlLapA*, *SlPI I* and *SlPI II* in the TRV-SlMKK2- and TRV-SlMKK4-infiltrated plants were significantly reduced, showing >90% of reduction, as compared with those in the TRV-GUS-infiltrated control plants, at 24 hr after infection of *B. cinerea* (Figure 6B). These results indicate that silencing of *SlMKK2* and *SlMKK4* attenuates significantly the expression of both SA signaling- and JA/ET signaling-regulated defense genes in tomato plants upon infection of *B. cinerea*.

### Transient expression of *SlMKK2*/*SlMKK4* in *Nicotiana benthamiana* activated defense responses against *B. cinerea*

**Figure 7:**
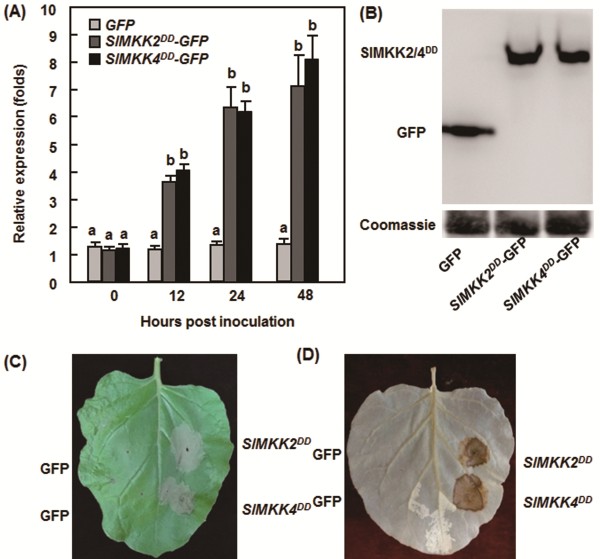
**Transient expression of constitutively active phosphomimicking forms *SlMKK2***^***DD***^**and *SlMKK4***^***DD***^**in *Nicotiana benthamiana *resulted in hypersensitive response and accumulation of ROS. A**. Expression of *SlMKK2*^*DD*^ and *SlMKK4*^*DD*^. Gene expression was analyzed by qRT-PCR and relative expression levels were calculated by comparing with the corresponding values at 0 hr (as a control) after infiltration. **B**. Proteins of SlMKK2^DD^ and SlMKK4^DD^. Leaf samples were harvested 48 hr after infiltration and total soluble protein extracts were prepared. Proteins were separated by SDS–PAGE and analyzed by immunoblotting using a GFP-specific antibody. Total proteins showing equal loading were examined by Coomassie staining. **C**. HR-like cell death. Photo was taken 48 hr after infiltration. **D**. Accumulation of H_2_O_2_. Detection of H_2_O_2_ was performed by DAB staining at 48 hr after infiltration. Data presented in **(A)** are the means ± SD from three independent experiments and different letters above the columns indicate significant differences at *p* < 0.05 level.

**Figure 8:**
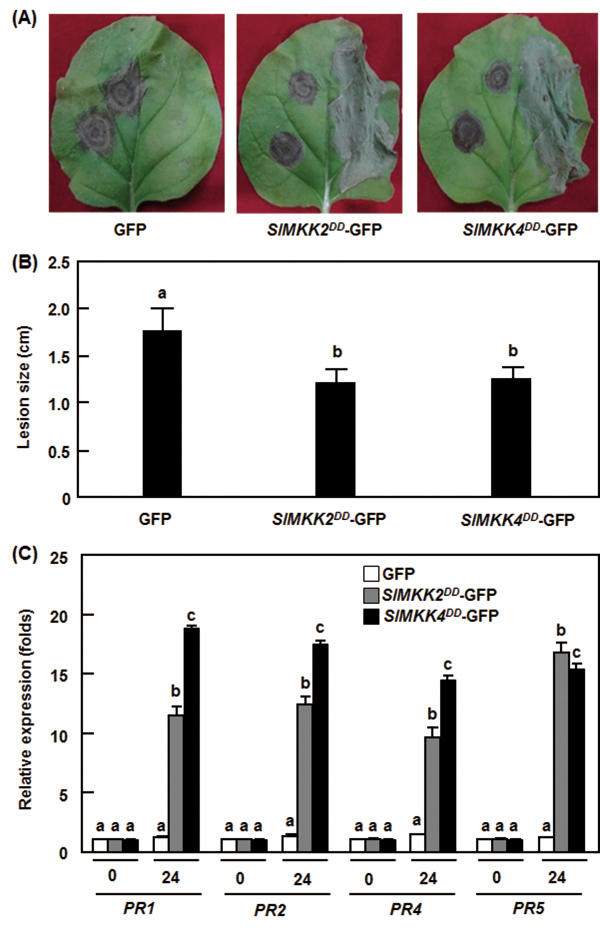
**Transient expression of constitutively active phosphomimicking forms *SlMKK2***^***DD ***^**and *SlMKK4***^***DD ***^**in *Nicotiana benthamiana *resulted in increased disease resistance against *B. cinerea*.** Disease symptom **(A)**, lesion size **(B)** and expression of defense genes **(C)** in *SlMKK2*^*DD*^- and *SlMKK4*^*DD*^-infiltrated plants. Opposite part of the leaves infiltrated with *SlMKK2*^*DD*^ and *SlMKK4*^*DD*^ constructs was inoculated by dropping spore suspension (2 × 10^5^ spores/mL) of *B. cinerea*. Lesion sizes were measured at 5 days after inoculation on a minimum of 10 leaves in each experiment. Expression of defense genes was analyzed by qRT-PCR at indicated times and relative expression levels were calculated by comparing with the corresponding values at 0 hr (as a control) after inoculation. Data presented in **(C)** are the means ± SD from three independent experiments and different letters above the columns indicate significant differences at *p* < 0.05 level.

To further confirm the function of *SlMKK2* and *SlMKK4* in resistance to *B. cinerea*, we examined whether overexpression of *SlMKK2* or *SlMKK4* can confer an increased resistance to *B. cinerea*. In our initial experiments, we were unable to observe typical HR when transiently expressed the wild types of *SlMKK2* and *SlMKK4* genes in *Nicotiana benthamiana* leaves (data not shown). This differed from previous observations that transient expression of *SlMKK2* and *SlMKK4* in tomato and *N. benthamiana* leaves resulted in HR production [41]. Considering that SlMKK2 and SlMKK4 are components of the MAPK cascades that require protein phosphorylation for their biochemical functions, we thus generated constitutively active phosphomimicking forms of SlMKK2 and SlMKK4, SlMKK2^DD^ and SlMKK4^DD^, by replacing the conserved Ser/Thr residues in the activation loop ((S/T)XXXXX(S/T)) with Asp [50]. When transiently expressed in *N. benthamiana* leaves, high levels of *SlMKK2*^*DD*^ and *SlMKK4*^*DD*^ expression and the SlMKK2^DD^-GFP and SlMKK4^DD^-GFP fusion proteins were detected (Figure 7A and B). Transient expression of either *SlMKK2*^*DD*^ or *SlMKK4*^*DD*^ resulted in a typical and strong HR and significant accumulation of H_2_O_2_ in the infiltrated areas of *N. benthamiana* leaves 48 hr after infiltration (Figure 7C and D), indicating that an activated phosphorylation status of SlMKK2 and SlMKK4 is necessary for their biochemical functions. We infiltrated the SlMKK2^DD^ and SlMKK4^DD^ constructs into one side of the *N. benthamiana* leaves for transient expression and then inoculated the opposite side of the leaves with spore suspension of *B. cinerea* 48 hr after infiltration. In our experiments, tissues collapse due to strong HR was always observed in the SlMKK2^DD^- and SlMKK4^DD^-infiltrated halves of the leaves (Figure 8A). In disease assays, the lesions on the opposite halves of the leaves from the SlMKK2^DD^- and SlMKK4^DD^-infiltrated *N. benthamiana* plants were significantly smaller than that in eGFP vector-infiltrated control plants (Figure 8A), leading to approximately 40% of reduction in lesion size, at 5 days after inoculation with *B. cinerea* (Figure 8B). To examine whether the enhanced disease responses induced by transient expression of *SlMKK2*^*DD*^ and *SlMKK4*^*DD*^ were linked to change in the regulation of defense genes. We also analyzed and compared the expression of some selected defense genes in leaves of the eGFP vector-, SlMKK2^DD^- and SlMKK4^DD^-infiltrated plants. As shown in Figure 8C, the expression levels of *PR1*, *PR2*, *PR4* and *PR5* in the SlMKK2^DD^- and SlMKK4^DD^-infiltrated plants were significantly increased at 24 h after infiltration, showing 10–24 folds of increases over those in the eGFP vector-infiltrated plants (Figure 8C). These data demonstrate that phosphorylated SlMKK2 and SlMKK4 positively regulate defense response against *B. cinerea* and that phosphorylation of SlMKK2 and SlMKK4 is required for their functions in plant immunity.

## Discussion

The MAPK cascades, as an important module that mediates the transduction and amplification of the environmental signals from plasma membrane-localized receptors/sensors into plant cells, play critical roles in defense responses against pathogen attack (for reviews, see [11,15,16,32,33]). Regarding a large body of evidence on the functions and mechanisms of the MAPK cascades in plant innate immune responses (i.e. PTI and ETI) against biotrophic/hemibiotrophic pathogens, the function of the MAPK cascades in defense response against necrotrophic fungal pathogens, which have distinct infection styles from that of biotrophic pathogens [48,51], is relatively limited. When searched in the literatures, only a few of studies have examined phenotypically using loss-of-function and gain-of-function approaches the functions of individual component of MAPK cascades, i.e. AtMPK3, AtMPK4 and AtMKK2, in resistance to necrotrophic fungi such as *B. cinerea* and *Alternaria brassicicola*[20,21,52,53]. We previously demonstrated that the tomato SlMPK4, a homolog of AtMPK4, is required for resistance to *B. cinerea*[40]. In the present study, we showed that two tomato MKKs, SlMKK2 and SlMKK4, are also required for resistance to *B. cinerea* and function as positive regulators of defense response against *B. cinerea*. Our findings provide new insights into the understanding of the molecular mechanism for the MAPK cascades in regulating tomato immune response against necrotrophic fungal pathogens.

Four SlMKKs were previously identified [41]. In the present study, we identified the fifth SlMKK, SlMKK5, which belongs to Group B of plant MKKs [13] and seems to be a close homologue of Arabidopsis AtMKK3 (Figure 1). Our identification of SlMKK5 led to a total of five members for the tomato MKK family, which fall into different groups of plant MKKs [13]. Surprisingly, the number of the SlMKK family is obviously lesser than those in other plant species such as Arabidopsis (10 AtMKKs) [13], rice (8 OsMKKs) [14], soybean (11 GmMKKs) [54]; popular (13 PtMKKS) [14]; apple (9 MdMKKs) [55], canola (7 BnaMKKs) [56] and *Brachypodium distachyon* (12 BdMKKs) [57]. For instance, two close homologues of MKK2 exist in Arabidopsis and rice genomes (i.e. AtMKK4/AtMKK5 and OsMKK4/OsMKK5). However, only one MKK2 was found in three *Nicotiana* species (common tobacco, *N. benthamiana* and *N. attenuate*) [58] and in tomato (Figure 1). Relatively fewer members of the SlMKK families in tomato and probably in other Solanaceae plants may be due to species-specific diversification during evolutionary history. On the other hand, the smaller number of the SlMKK family in tomato also suggests that the tomato SlMKK proteins may have evolved to play pleiotropic roles in diverse biological processes.

Activity of the MAPK cascades can be regulated at both transcriptional level and post-translational level. Transcriptional regulation of expression of genes for *MKKs* was reported in a range of plants upon different biotic and abiotic stress. For instance, the Arabidopsis *AtMKK3*, cotton *GhMKK4* and *GhMKK5* and *N. attenuata NaMKK1* were recently shown to be induced by different pathogens (i.e. *P. syingae* pv. *tomato* DC3000, *Rhizoctnia solani*, *Fusariun oxysporum* f.sp. *vasinfectum*), defense signaling molecules (i. e. SA, JA and ethephon) and herbivores [58-61]. Similarly, we also found in this study that the five tomato *SlMKK* genes are responsive to *B. cinerea* and that *SlMKK2* and *SlMKK4* can be induced rapidly and strongly after infection of *B. cinerea* (Figure 2). The inducibility of the expressions of *SlMKK2* and *SlMKK4* by SA, JA and ACC (Figure 3) indicates that these two *SlMKKs* may be involved in both the SA- and JA/ET-mediated signaling pathways that activate defense responses against different types of pathogens. The significance of the transcriptional regulation of MKKs is also supported by several observations that overexpression of wild type forms of the *MKK* genes in transgenic plants or increased expression in activation-tagged mutant plants can result in altered resistance against a range of pathogens [60-62]. However, biochemical activation of the MAPK cascades at the post-translation level, which involves phosphorylation by upstream signals, is critical to their functions as signaling modules. To this regard, further biochemical experiments are required to examine whether SlMKK2 and SlMKK4 and their involved MAPK cascades are activated in tomato plants upon infection of *B. cinerea*.

In our VIGS-based phenotyping of all five SlMKKs, no any altered response of the *SlMKK1*-, *SlMKK3*- or *SlMKK5*-silenced plants to *B. cinerea* was observed (Figure 4). The Arabidopsis AtMKK2, a closely related homolog of SlMKK1 (Figure 1), has been shown to function as a negative regulator of immune response against biotrophic/hemibiotrophic pathogens [28-30] and overexpression of constitutively active form AtMKK2^EE^ resulted in enhanced susceptibility to *A. brassicicola*[53]. AtMKK2 has a redundant function with AtMKK1 and both AtMKK1 and AtMKK2 act upstream of AtMPK4 in the MEKK1-MKK1/2-MPK4 cascade [29,63]. Silencing of *SlMPK4*, a homolog of AtMPK4, resulted in reduced resistance to *B. cinerea*[40]. Surprisingly, silencing of *SlMKK1*, a possible MKK that acts upstream of SlMPK4, did not affect resistance to *B. cinerea* (Figure 4). The Arabidopsis AtMKK3, closely related to SlMKK5 (Figure 1), has been demonstrated to participate in a partial MAPK cascade that plays an important role in regulating expression of a set of JA-responsive genes, which are involved in JA-mediated defense responses [59,64]. However, in our study, silencing of *SlMKK5* also did not affect the resistance to *B. cinerea* (Figure 4), similar to a previous observation that silencing of *SlMKK3* did not affect resistance to *Xanthomonas campestris* pv. *vesicatoria*, the causal agent of bacterial spot disease on tomato [65]. The phylogenetically related members of the SlMKK3 from other plants have not been functionally analyzed for their biological functions, but the rice OsMEK1 and maize ZmMEK1, closely related to SlMKK3 (Figure 1) [41] were shown to be involved in primary roots and abiotic stress response [66,67]. Thus, it is possible that SlMKK3 may not be involved in disease resistance to *B. cinerea* (Figure 4). Regarding to the SlMKK1 and SlMKK5, however, their involvement in resistance to *B. cinerea* and to other pathogens cannot be ruled out before the disease phenotypes in plants with overexpression of the constitutively active phosphomimicking forms of SlMKK1 and SlMKK5 are carefully examined.

The function of SlMKK2 and SlMKK4 in resistance to *B. cinerea* is supported by several observations presented in this study. Firstly, silencing of *SlMKK2* and *SlMKK4* resulted in reduced resistance to *B. cinerea*, as shown in detached leaf disease assays and whole plant disease assays (Figure 4 and Figure 5). SlMKK2 is closely related to Arabidopsis AtMKK4 and AtMKK5 (Figure 1). The reduced resistance to *B. cinerea* in the *SlMKK2*-silenced plants is somewhat similar to the observation that the Arabidopsis *mpk3* plants showed reduced basal resistance to *B. cinerea*[20,21], although there is no direct experimental evidence indicating whether mutations in AtMKK4 and AtMKK5, two upstream MKKs of AtMPK3 [17,19], affect basal resistance to *B. cinerea*. Meanwhile, it was found that silencing of *NbMKK1*, closely related to SlMKK4 (Figure 1), attenuated resistance against a nonhost pathogen *Pseudomonas cichorii*[68]. Previous studies have shown that silencing of *SlMKK2* resulted in reduced resistance against *P. syringae* pv. *tomato* and *X. campestris* pv. *vesicatoria*[35,65], indicating that SlMKK2 also plays a role in disease resistance against other pathogens.

Secondly, silencing of *SlMKK2* and *SlMKK4* attenuated defense responses, i.e. generation of ROS and expression of defense genes (Figure 6), induced by infection of *B. cinerea*. In our study, silencing of *SlMKK2* or *SlMKK4* resulted in significant accumulation of ROS after infection of *B. cinerea* (Figure 6A), consistent with the increased disease severity (Figures 4 and 5). This is in agreement with a general hypothesis that ROS accumulated during the late stage directly benefits the establishment of infection by *B. cinerea*[48]. Several studies have demonstrated that *B. cinerea* induces the generation of ROS in plants to the benefit of the pathogen [69-71]. Comparison of the kinetics of ROS accumulation between the abscisic acid-deficient *sitiens* tomato mutant plants (highly resistant to *B. cinerea*) and the susceptible wild type plants after infection with *B. cinerea* revealed that timing of ROS accumulation is critical to its role in disease development [72]. H_2_O_2_ accumulation in wild-type tomato plants started at 24 hr while H_2_O_2_ accumulation in *sitiens* plants was observed as early as 4 hr after inoculation [72]. In our study, significant accumulation of H_2_O_2_ at relatively late stage (24 hr after inoculation) in the *SlMKK2*- and *SlMKK4*-silenced plants may start to initiate cell death in the site of infection and thus facilitate growth and infection of *B. cinerea*. This is partially supported by the significant difference of fungal growth in the TRV-SlMKK2- and TRV-SlMKK4-infiltrated plants and the TRV-GUS-infiltrated plants at 24 hr after inoculation (Figure 5B). Therefore, ROS accumulation in *B. cinerea*-infected tissues of plants may contribute differentially to disease development and disease resistance response depending on the timing kinetics of ROS production and accumulation as a facilitator of cell death may promote susceptibility, but early ROS may induce resistance mechanisms [48]. On the other hand, expression of *SlPRP2* and *SlPR1b*, regulated by the SA-mediated signaling pathway [49], and *SlLapA*, *SlPI I* and *SlPI II*, regulated by the JA/ET-mediated signaling pathway [49], were significantly decreased in the *SlMKK2*- and *SlMKK4*-silenced plants after infection of *B. cinerea* (Figure 6B), indicating that SlMKK2 and SlMKK4 may be involved in both SA - and JA/ET-mediated signaling pathways in tomato plants upon infection of *B. cinerea*. This is partially supported by the observations that the Arabidopsis AtMPK3 and AtMPK6, downstream MAPK of AtMKK4 and AtMKK5, closely related to SlMKK2 (Figure 1), are implicated in *B. cinerea*-induced ET biosynthesis [22] and that overexpression of AtMKK7, related to SlMKK4 (Figure 1), leads to elevated levels of SA [62].

Thirdly, transient expression of the constitutively active phosphomimicking forms *SlMKK2*^*DD*^ and *SlMKK4*^*DD*^ in *N. benthamiana* plants led to HR-like cell death, overproduction of ROS, enhanced resistance to *B. cinerea* and upregulated expression of defense genes (Figures 7 and 8). These phenotypes are consistent with the observations that transient expression of constitutively active forms of Arabidopsis *AtMKK4* or tobacco *NtMEK2* resulted in PCD and enhanced resistance to *B. cinerea*[17,19]. Generally, HR-like cell death, probably caused by ROS accumulated during the late infection stage, facilitates colonization of plants by *B. cinerea*[69,70]. However, the coincidence of HR-like cell death and enhanced resistance against *B. cinerea* in *N. benthamiana* plants transiently expressed the constitutively active phosphomimicking forms *SlMKK2*^*DD*^ and *SlMKK4*^*DD*^ may indicate that not all HR-like cell death is correlated with susceptibility to necrotrophic fungal pathogens like *B. cinerea*. This hypothesis is supported by recent observations that the control of cell death governs the outcome of the *Sclerotinia sclerotiorum*-plant interaction [73]. On the other hand, it was previously reported that expression of wild type forms of *SlMKK2* and *SlMKK4* in leaves of tomato and *N. benthamiana* plants caused typical PCD [41]. However, we failed to observe the appearance of PCD in leaves of *N. benthamiana* plants infiltrated with constructs of wild type of *SlMKK2* or *SlMKK4* (data not shown). This is similar to the observation for AtMKK3, whose overexpression in its wild type form did not affect the resistance to *P. syringae* pv. *tomato* DC3000 [59]. Interestingly, when the *SlMKK2*^*DD*^ or *SlMKK4*^*DD*^ construct was transiently expressed in one half of leaves, the opposite half of the same leaves showed enhanced resistance to *B. cinerea* and upregulated expression of defense genes upon infection of *B. cinerea* (Figure 8), indicating that SlMKK2 and SlMKK4 may have a systemic effect on activation of defense response. It was recently found that ectopic expression of *AtMKK7* in local tissues could induce disease resistance in systemic tissues, demonstrating a critical role for AtMKK7 in generating the systemic signal of SAR [62]. In our experiments, significant H_2_O_2_ accumulation due to transient expression of *SlMKK2*^*DD*^ or *SlMKK4*^*DD*^ construct in one half of the *N. benthamiana* leaves at 48 hr after infiltration, at the time when the opposite half of the same leaves was inoculated with *B. cinerea*, may mount the ROS generated during the early stage of infection. It is therefore possible that ROS generated in the half leaf that transiently expressed the *SlMKK2*^*DD*^ or *SlMKK4*^*DD*^ construct may trigger the generation of yet unknown systemic signal(s), which transduce and activate defense responses in the opposite half leaf.

## Conclusion

Tomato genome encodes five *SlMKK* genes and both of *SlMKK2* and *SlMKK4* can be induced by *B. cinerea*. Silencing of *SlMKK2* and *SlMKK4* resulted in reduced resistance to *B. cinerea*, increased accumulation of ROS and attenuated expression of defense genes after infection with *B. cinerea* in tomato. Transient expression of the constitutively active phosphomimicking forms *SlMKK2*^*DD*^ and *SlMKK4*^*DD*^ in *N. benthamiana* plants led to enhanced resistance to *B. cinerea* and elevated expression of defense genes. Our results demonstrated that both SlMKK2 and SlMKK4 function as positive regulators of defense response against *B. cinerea* in tomato.

## Methods

### Plant growth, treatments and disease assays

Tomato (*Solanum lycopersicum*) cv. Suhong 2003 was used for all experiments. Seeds were scarified on moist filter paper in Petri dishes for 3 days and the sprouted seeds were transferred into a mixture of perlite : vermiculite : plant ash (1:6:2). Tomato and *N. benthamiana* plants were grown in a growth room under fluorescent light (200 μE m^2^ s^−1^) at 22–24°C with 60% relative humidity in a 14 hr light/10 hr dark regime. For analysis of gene expression, 4-week-old tomato plants were treated by foliar spraying with 10 μM MeJA, 100 μM ACC, 100 μM SA or water as a control and samples were collected at indicated time points after treatment.

For disease assays, inoculation of *B. cinerea* was performed using spore suspension at spore density of 1 × 10^5^ spores/mL according to previously reported procedure [74]. Two different inoculation assays, whole plant inoculation and detached leaf inoculation, were used for different purposes. The whole plant inoculation assays were adapted to quantitatively analyze fungal growth *in planta*, whereas the detached leaf inoculation assays were used to quantitatively measure lesion sizes. In the whole plant inoculation assays, 4-week-old plants were inoculated by foliar spraying with spore suspension or buffer (as a mock-inoculation control). In detached leaf inoculation assays, fully expanded leaves from at least twelve individual plants from each treatment were inoculated by dropping a 5 μL of spore suspension onto leaf surface. The inoculated leaves and plants were kept in a humidity condition by covering with plastic film in trays or tans at 22°C to facilitate disease development. Leaf samples were collected from the whole plant inoculation assays at different time points after inoculation for analysis of gene expression and fungal growth *in planta*. Fungal growth was measured by qRT-PCR analyzing the transcript of *B. cinerea ActinA* gene as an indicative of fungal growth [75] using a pair of primers BcActin-F and BcActin-R (Table 1). Disease progress in the detached leaf inoculation assays was estimated by measuring the lesion sizes at time points as indicated.

**Table 1:** Primers used in this study for different purposes

**Primers**	**Sequences (5′-3′)**	**Size (bp)**
*Cloning of cDNA*		
SlMKK1-F	ATGAAGAAAGGATCTTTTGCAC	1074
SlMKK1-R	TTATAGCTCAGTAAGTGTTGCC	
SlMKK2-F	ATGCGACCAGCCGCCAACTCCA	1080
SlMKK2-R	TCAAGAAGAGGAGGAAAAATGA	
SlMKK3-F	ATGAAGACGGCGAAGCCATTGA	1065
SlMKK3-R	TTATCTTGGAAAATTTACTGGG	
SlMKK4-F	ATGGCCTTAGTTCGTGATCGCC	1008
SlMKK4-R	TTAGGTGGATTTCAAATCGATA	
SlMKK5-F	ATGGCTGGACTGGAGGAATTG	1548
SlMKK5-R	CTATTGAGTAATGAAAAGTTC	
*VIGS constructs*		
SlMKK1-VIGS-F	TGC TCTAGAGCAAAACCCCATTTGCCTGA	360
SlMKK1-VIGS-R	CCG GAGCTCCTGATGGCTGTATAACTGAA	
SlMKK2-VIGS-F	TGC TCTAGACAATCCAATACCATTATTCA	360
SlMKK2-VIGS-R	TCCCCCGGGAAGACGGACAGAATCCTCGT	
SlMKK3-VIGS-F	TGC TCTAGAGGGTTATTTGGGCGGTCCGT	360
SlMKK3-VIGS-R	CCG GAGCTCAATGTTCCAACCCATTTATG	
SlMKK4-VIGS-F	TGCTCTAGA ACTTGAAAAGCTTAAGGTTC	400
SlMKK4-VIGS-R	CCGCTCGAG ACGATTCACTAAAAGGTTCG	
SlMKK5-VIGS-F	TGC TCTAGAATGGCTGGACTGGAGGAATT	360
SlMKK5-VIGS-R	CCG GAGCTCCTGCCTTTTCTCCTTCTCAA	
*Transient expression*		
SlMKK2^DD^-GFP-F	GCTGTACAAGGGATCCATGCGACCTCTTCAACCACC	1080
SlMKK2^DD^-GFP-R	TAATTAACTCTCTAGATTAAGAAGAAAAATGAGGAG	
SlMKK4^DD^-GFP-2F	TGCTCTAGAATGGCCTTAGTTCGTGATCGCCG	1008
SlMKK4^DD^-GFP-2R	TCCCCCGGGTTAGGTGGATTTCAAATCGATAC	
*qRT-PCR*		
SlMKK1-RT-F	GGCCAATACCTTTGTCGGCACATA	148
SlMKK1-RT-R	TCCCTCGGGTGGTTTATATGGGAA	
SlMKK2-RT-F	AAGGTTCTACATCGTCCCACTGGA	100
SlMKK2-RT-R	TCTCGATCTCACGGCACATCTGAA	
SlMKK3-RT-F	AATGCTAGCCAGCTCTATGGGTCA	181
SlMKK3-RT-R	AGCTTGCTGGTCCTCTGACTGTAT	
SlMKK4-RT-F	CGCTAAGCAAGTGCTTGGTGGATT	158
SlMKK4-RT-R	TGCAAGGATCCAAAGTCCTTCCCA	
SlMKK5-RT-F	CCAGAACCTATCCTTTCCTCAAT	103
SlMKK5-RT-R	GATTTGCTGGCTTTATGTCTCTG	
SlActin-RT-F	CCAGGTATTGCTGATAGAATGAG	113
SlActin-RT-R	GAGCCTCCAATCCAGACAC	
SlPI I-RT-R	GTTGTACAAATGCCTGTGGTGAC	135
SlPI I-RT-R	GGTAAGAGTACATGAAGAGATGC	
SlPI II-RT-F	CATCTTCTGGATTGCCCA	106
SlPI II-RT-R	ACACACAACTTGATGCCCAC	
SlLapA-RT-F	GGGACTAATGATGTTTGGAA	109
SlLapA-RT-R	GTGGCAATTTTATTTAGGCA	
SlPR1b-RT-F	TTTCCCTTTTGATGTTGCT	96
SlPR1b-RT-R	TGGAAACAAGAAGATGCAGT	
SlPR-P2-RT-F	CGATCTAAATTGATTTCATAGTACG	116
SlPR-P2-RT-R	TCGTGAAGGATATACAAAATACA	
BcActin-RT-F	CGTCACTACCTTCAACTCCATC	107
BcActin-RT-R	CGGAGATACCTGGGTACATAGT	
NbActin-RT-F	ACCAGATTAATGAGCCCAAGAG	97
NbActin-RT-R	CCAACAGGGACAGTACCAATAC	
NtPR1-RT-F	CCGTTGAGATGTGGGTCAAT	100
NtPR1-RT-R	CGCCAAACCACCTGAGTATAG	
NtPR2-RT-F	CAACCCGCCCAAAGATAGTA	98
NtPR2-RT-R	TGGCTAAGAGTGGAAGGTTATG	
NtPR4-RT-F	GGATGATGTTGACAGCAGAGA	116
NtPR4-RT-R	GTAGGACACGAGGTAGGTATCA	
NtPR5-RT-F	GCTCGATTACGTCTTGTCTCTC	104
NtPR5-RT-R	CTCTAGCATGGTGGATTGACTT	

### Extraction and treatment of total RNA

Extraction of total RNA from leaf samples by Trizol reagent and elimination of DNA in RNA samples with PrimeScript RT reagent Kit With gDNA Eraser (Takara, Dalian, China) were performed according to the manufacturer’s instructions. Total RNA samples obtained were stored at −80°C until used.

### Cloning of *SlMKKs* and construction of VIGS vectors

First-strand cDNA synthesis was performed using the AMV reverse transcriptase (Takara, Dalian, China) using oligo d(T) primer according to the manufacturer’s instructions. The coding sequences for *SlMKKs* were amplified using gene-specific primers (Table 1) designed based on available full-length cDNAs or predicted cDNAs and confirmed by cloning and sequencing. Fragments of 300–400 bp in sizes for *SlMKKs* were amplified using gene-specific primers (Table 1) from sequenced plasmids and cloned into TRV2 vector [47], yielding TRV2-SlMKK1-5. These constructs were introduced into *Agrobacterium tumefaciens* strain GV3101 by electroporation using GENE PULSER II Electroporation System (Bio-Rad Laboratories, Hercules, CA, USA).

### VIGS assays

Agrobacteria carrying TRV2-GUS (control) and TRV2-SlMKK1-5 plasmids were grown in YEP medium (50 μg/mL rifampicin, 50 μg/mL kanamycin and 25 μg/mL gentamicin) for 24 hr with continuous shaking at 28°C. Cells were centrifuged and resuspended in infiltration buffer (10 mM MgCl_2_, 10 mM MES, 200 μM acetosyringone, pH5.7). Agrobacteria carrying TRV2-GUS or TRV2-SlMKK1-5 were mixed with agrobacteria carrying TRV1 in a ratio of 1:1 and adjusted to OD_600_ = 1.5. The mixed agrobacteria suspension was infiltrated into the abaxial surface of 2-week-old seedlings using a 1 mL needleless syringe. Efficiency of the silencing protocol was examined using a tomato *PDS* gene as a marker of silencing in tomato plants according to the protocol described previously [47].

### Transient expression in *N. benthamiana*

Constitutively active phosphomimicking forms of SlMKK2 and SlMKK4, SlMKK2^DD^ and SlMKK4^DD^, respectively, were generated by replacing the conserved Thr (Thr-215 for SlMKK2 or Thr-216 for SlMKK4) and Ser (Ser-221 for SlMKK2 or Ser-222 for SlMKK4) residues between the kinase subdomains VII and VIII with Asp using the QuikChange site-directed mutagenesis kit (Stratagene) as described previously [50]. The mutated sequences in SlMKK2^DD^ and SlMKK4^DD^ were confirmed by sequencing and cloned into pFGC-Egfp vector to make SlMKK2^DD^-GFP and SlMKK4^DD^-GFP fusion constructs. The recombinant plasmids pFGC-SlMKK2^DD^-GFP, pFGC-SlMKK4^DD^-GFP and pFGC-Egfp were transformed into *A. tumefacies* GV3101. Agrobacteria carrying different constructs were grown overnight in YEP medium (50 μg/mL rifampicin, 50 μg/mL kanamycin and 25 μg/mL gentamicin), collected by centrifugation and resuspended to OD_600_ of 0.8 in infiltration buffer (10 mM MgCl_2_, 10 mM MES, 200 μM acetosyringone, pH5.7). Fully expanded leaves of 4-week-old *N. benthamiana* were infiltrated with agrobacterial suspension as described before [50] and leaf samples were collected at 48 hr after infiltration for disease assays and for physiological, biochemical and molecular analyses.

### Western blotting

Leaf discs were ground in 200 μL extraction buffer (4 M uera, 100 mm DTT), followed by addition of 100 μL loading buffer. The samples were boiled for 5 min and subsequently centrifuged at 10000 g for 10 min at 4°C. Proteins in 20 μL of the supernatant were separated on a 15% SDS-PAGE gel and transferred onto nitrocellulose by wet electroblotting. Detection of GFP was performed using a mouse monoclonal GFP antibody (1:1000 dilution) (No. M1210-1, Huaan Company, Hangzhou, China) and a peroxidase-conjugated antimouse antibody (1:8000 dilution (No. HA1008, Huaan Company, Hangzhou, China) according to the manufacturer’s instructions. Proteins in SDS-PAGE gel were detected by an ECL Plus detection system (Huaan Company, Hangzhou, China).

### qRT-PCR analysis of gene expression

For gene expression analyses, qPCR was performed with three independent biological replicates using SYBR PrimeScript RT-PCR Kit (TaKaRa, Dalian, China) in a 25 μL volume on a CFX96 Real-time System (Bio-Rad, Hercules, CA, USA). A tomato actin gene was used as an internal control for normalization of the data obtained. Relative expression was calculated using 2^–△△CT^ method.

### Detection of ROS

Detection of H_2_O_2_ was performed by 3, 3-diaminobenzidine (DAB) staining [76]. Leaf samples were collected from inoculated tomato plants at 24 h after inoculation or *N. benthamiana* plants at 48 h after infiltration for transient expression. Leaves were dipped into DAB solution (1 mg/ml, pH3.8) and incubated for 8 hr in dark at room temperature. The DAB-treated leaves were removed, placed into acetic acid/glycerol/ethanol (1:1:1, vol/vol/vol), and boiled for 5 min in a water bath, followed by several changes of the solution. Subsequently, the leaves were maintained in 60% glycerol and accumulation of H_2_O_2_ was visualized using a digital camera.

### Statistical analysis

All experiments were repeated independently for at least three times. Data obtained were subjected to statistical analysis according to the Student’s *t*-test and the probability values of *p* < 0.05 were considered as significant between different treatments.

## Competing interests

The authors declare that they have no competing interests.

## Authors’ contributions

XL, YZ, LH, ZO, YH and HZ carried out most of the experiments. DL performed bioinofrmatics analysis. XL and FS designed the experiments. FS and XL wrote the paper. All authors read and approved the final manuscript.
